# Evidence of a Large Diversity of *N*-acyl-Homoserine Lactones in Symbiotic *Vibrio fischeri* Strains Associated with the Squid *Euprymna scolopes*

**DOI:** 10.1264/jsme2.ME18145

**Published:** 2019-02-13

**Authors:** Léa Girard, Elodie Blanchet, Didier Stien, Julia Baudart, Marcelino Suzuki, Raphaël Lami

**Affiliations:** 1 Sorbonne Universités, UPMC Univ. Paris 06, CNRS, Laboratoire de Biodiversité et Biotechnologies Microbiennes (LBBM), Observatoire Océanologique F-66650 Banyuls/Mer France

**Keywords:** Quorum sensing, *N*-acyl-homoserine lactone, *Vibrio fischeri*, *Euprymna scolopes*

## Abstract

*Vibrio fischeri* possesses a complex AHL-mediated Quorum-sensing (QS) system including two pathways, LuxI/R (3-oxo-C6-HSL and C6-HSL) and AinS/R (C8-HSL), which are important for the regulation of physiological traits. Diverse QS-dependent functional phenotypes have been described in *V. fischeri*; however, AHL diversity is still underestimated. In the present study, we investigated AHL diversity in five symbiotic *V. fischeri* strains with distinct phenotypic properties using UHPLC-HRMS/MS. The results obtained (1) revealed an unexpectedly high diversity of signaling molecules, (2) emphasized the complexity of QS in *V. fischeri*, and (3) highlight the importance of understanding the specificity of AHL-mediated QS.

Quorum sensing is a phenomenon that was originally observed in *Vibrio fischeri* (26). The bioluminescence mechanism that *V. fischeri* triggers at high cell density led to the discovery of *N*-acyl-homoserine lactones (AHL). These initial investigations resulted in the identification of the *N*-3-oxo-hexanoyl-homoserine lactone (3-oxo-C6-HSL) and the description of the now well-known QS system LuxI/LuxR (6, 7). The AinS/AinR system was subsequently identified and a second autoinducer, *N*-octanoyl-homoserine lactone (C8-HSL) was characterized (11, 21). This autoinducer is involved in early colonization factors needed for the settlement of *V. fischeri* in the light organ (15, 24). Since these initial studies, many others have revealed a wide genetic and phenotypic diversity in symbiotic strains of *V. fischeri*. Heterogeneous phenotypes have been observed, such as variants in bioluminescence intensity, motility, growth rate, siderophore production, colony pigmentation, and competence in colonizing the light organ (S and D strains). These findings support the existence of intraspecific differences among *V. fischeri* strains related to host biology (2, 15, 34). However, it currently remains unclear whether distinct AHL production patterns are associated with these phenotypic variations.

Due to the recent development of more sensitive methods in analytical chemistry, the detection of more diverse AHL molecules is possible (12, 28). The AHL molecule consists of a hydrophilic homoserine lactone ring moiety and a hydrophobic acyl side chain. The latter may lead to a wide diversity of molecules through variations in length, substitutions at the beta-position, and the level of saturation (9). These different features result from the specificity of the AHL synthase for its substrates and influence interactions with the corresponding receptor (4, 16). Considering that genetic diversity, metabolism, and environmental factors are likely to modulate AHL production (13, 17, 30, 33), it seems essential to characterize the AHL diversity among *V. fischeri* strains in order to determine if their different genetic and phenotypic characteristics are related to distinct AHL production patterns.

The aims of the present study were to establish whether *V. fischeri* strains with different phenotypes exhibit common or distinct chemical languages and to investigate the possible link between AHL production and genetic diversity (whole genome, AHL synthases, and receptors). Our work was based on symbiotic isolates previously collected in *Euprymna scolopes* squids from different geographical locations, which were genotyped (VfRep, PCR fingerprints, and whole-genome sequencing) and phenotyped, particularly for their capacity to colonize the light organ (S and D strains) (2, 24, 25, 34). Two phenotypes were observed in co-colonization experiments: a “Dominant” phenotype (D-type strain; more than 50% of the squid is colonized by this strain only) and a “Shared” phenotype (S-type strains; the two strains colonize the squid equally) (2). This study expands on an earlier investigation that identified AHLs from *V. tasmaniensis* LGP32 using a novel Ultra-High-Performance Liquid Chromatography followed by High-Resolution tandem Mass Spectrometry (UHPLC-HRMS/MS) methodology to include a sub-selection of *V. fischeri* strains (ES213, KB2B1, KB4B5, MB13B1, and ES114) (12).

Five strains of *V. fischeri* were selected for their colonization capacities (D-*type*: ES213 and KB2B1, or S-*type*: ES114, KB4B5, and MB13B1) and the sampling location of the host squids (Kaneohe Bay, KB: ES114, KB4B5, and KB2B1, or Maunalua Bay, MB: ES213 and MB13B1; 1, 34). Isolates were cultivated in Luria-Bertani (LB) salt medium at 28°C for 24 h under shaking (120 rpm) using the cultivation conditions described by Bongrand *et al*. (2). Regarding each strain, 3 L of culture was extracted with ethyl acetate when cultures reached an OD_600_ ~1.5. Ethyl acetate extracts were treated prior to analysis using solid-phase extraction (Phenomenex Strata C18, 55 1.1 m). The stationary phase (SPE cartridge, Solid-Phase Extraction) was activated with 100 mL of acetonitrile and elution was performed with 50 mL of acetonitrile to eliminate the most lipophilic metabolites. Eluates were evaporated to dryness and dissolved in DMSO. The pH of culture and extracts did not exceed 7.5 throughout the entire procedure.

Extracts were fractionated on a Phenomenex Luna C18 column (5 mm, 250×21.20 mm, 100 Å) using a separative HPLC system with 2 Varian Prep Star pumps, a manual injector, Dionex Ultimate 3000 RS variable wavelength detector, and Dionex Ultimate 3000 fraction collector. The mobile phase consisted of HPLC grade H_2_O and CH_3_CN (70:30 for 3 min, followed by a 12-min linear gradient from 70:30 to 0:100 and then 100% CH_3_CN for 10 min) and pumped at a flow rate of 20 mL min^−1^. Eluents were monitored at 214, 254, 274, and 280 nm and were collected between 3 and 25 min (1 fraction min^−1^, 22 fractions). The solvent was removed with a HT-4X system (Genevac, Biopharma Technologies France, Lyon, France), and each fraction was dissolved in 100 μL DMSO and then diluted at 1/4 with LB medium (v/v) for biosensor tests. Positive fractions were further analyzed by UHPLC-HRMS/MS.

Prior to injection, fractions were diluted to 1 mg mL^−1^ and 5 μL was injected. UHPLC-MS analyses were performed with a Dionex Ultimate 3000 UHPLC-HESI HRMS Q-Exactive focus system (Thermo Fisher Scientific, Waltham, MA, USA) controlled by Xcalibur software. The column was Hypersil GOLD C18 (2.1 mm×150 mm) with a particle size of 1.9 μm (Thermo Scientific). Experimental settings were identical to those described in our previous study (12). MS and MS/MS profiles were recorded alternating between full scan and All Ion Fragmentation (AIF) mode to determine molecular weights and identify chromatographic peaks generating fragment ions at m/z 102.055. AHL identifications were confirmed by a second analysis using Selected Ion Monitoring (SIM) and a dd-ms2 mode. The presence of the daughter ion with 102.055 m/z and at least one other daughter ion (at m/z 84.045, 74.060, or 56.050; characteristic of the lactone ring; 12, 28) were needed to confirm AHL identification. This confirmation was supported by the analysis of 27 commercially available AHL standards with different acyl chains lengths, substitutions (hydroxy or oxo group), and unsaturation.

We investigated AHL diversity using the non-targeted UHPLC-HRMS/MS method previously described by Girard *et al*. (12), which enables AHL detection based on MS/MS fragmentation patterns and is supported by the analysis of 27 AHL standards (Supplementary information, Table S1). To the best of our knowledge, this is the first study that inventories and compares AHL diversity across different *V. fischeri* symbiotic strains.

An initial analysis was conducted to detect AHL molecules by searching for molecular weights and retention times of parent ions generating a fragment ion at m/z 102.0555 corresponding to the lactone ring fragment. A second analysis was performed to study the MS/MS spectrum and identify four fragment ions at *m/z* 102.055, 84.045, 74.061, and 56.050, which are specific to the homoserine lactone (HSL) moiety (28). A comparison with the 27 different commercial standards enabled the direct identification of 10 AHLs: C6-HSL (*N*-hexanoyl-homoserine lactone), 3-oxo-C6-HSL (*N*-3-oxo-hexanoyl-homoserine lactone), C7-HSL (*N*-heptanoyl-homoserine lactone), C8-HSL (*N*-octanoyl-homoserine lactone), 3-OH-C8-HSL (*N*-3-hydroxy-octanoyl-homoserine lactone), 3-oxo-C8-HSL (*N*-3-oxo-octanoyl-homoserine lactone), C9-HSL (*N*-nonanoyl-homoserine lactone), C10-HSL (*N*-decanoyl-homoserine lactone), 3-OH-C10-HSL (*N*-3-oxo-decanoyl-homoserine lactone), and 3-OH-C12-HSL (*N*-3-hydroxy-dodecanoyl-homoserine lactone). The analysis of AHL standards was also used to build retention time (Rt) curves for Cx-HSL, OH-Cx-HSL, and oxo-Cx-HSL according to their acyl side chain lengths. The curves presented R^2^>0.99 for the three AHL types, and allowed the prediction of AHL retention times under our chromatographic conditions (Table 1 and Fig. S1). Two unusual AHLs with precursor masses and/or retention times that did not correspond with the standard AHLs were also detected by this method. 3-OH-C9-HSL (*N*-3-hydroxy-nonanoyl-homoserine lactone) and oxo-C16:2-HSL were confirmed by the presence of at least two of the fragment ions cited above and/or a retention time corresponding to the prediction curves (Table 1, S2, and S3). A structural elucidation by Nuclear Magnetic Resonance spectroscopy (NMR) would have been enough to determine the exact position of the oxo-C16:2-HSL unsaturation, however, because AHLs are produced at very low concentrations and NMR requires large amounts of pure compounds this analysis was not fulfilled in the present study.

Purohit *et al*. (29) recently reported the presence of six AHLs in strain ES114 (C4-HSL, 3-oxo-C6 HSL, C6-HSL, 3-OH-C8-HSL, C8-HSL, and C10-HSL), and we herein identified five additional AHLs for the same strain (C7-HSL, C9-HSL, 3-OH-C9-HSL, 3-OH-C10-HSL, and 3-OH-C12-HSL; Fig. 1). However, we did not recover C4-HSL or 3-oxo-C6-HSL because the limit of detection (LOD) of our UHPLC-HRMS/MS method for these two molecules was markedly beyond previously detected concentrations. Under similar culture conditions, Purohit *et al*. (29) detected a C4-HSL concentration of 70 nmol L^−1^, while our LOD exceeded 500 nmol L^−1^. Furthermore, Stabb *et al*. (32) detected a 3-oxo-C6-HSL concentration of 0.1 nmol L^−1^, while our LOD was equal to 10.90 nmol L^−1^. The two AHL synthases, LuxI and AinS, were initially reported to produce 3-oxo-C6-HSL and C8-HSL, respectively. In previous studies using similar culture conditions, 3-oxo-C6-HSL was produced at markedly lower quantities (between 0.01 and 0.2 nmol L^−1^; 32) than C8-HSL (between 2 and 1,100 nmol L^−1^; 23, 29). Therefore, it was not surprising that we detected C8-HSL in all the strains.

Besides the seven AHLs common to all strains (C6-HSL, C7-HSL, C8-HSL, 3-OH-C8-HSL, C9-HSL, 3-OH-C10-HSL, and 3-OH-C9-HSL), we identified three different AHL production patterns among our five strains of *V. fischeri*. Strains ES213, KB2B1, KB4B5, and ES114 were identified as producers of two additional AHLs (3-OH-C12-HSL and C10-HSL), oxo-C16:2-HSL was only detected in S*-type* strains (ES114, KB4B5, and MB13B1), and 3-oxo-C6-HSL and 3-oxo-C8-HSL were only detected in MB13B1 (Fig. 1). Therefore, this strain appears to produce larger quantities of 3-oxo-C6-HSL than the other four strains under identical culture conditions (23, 29, 32).

We examined the relationship between the genetic diversity of *V. fischeri* strains and their corresponding AHL production patterns. It is important to note that strains KB4B5 and ES114 were phylogenetically distinct based on whole-genome comparisons, but showed the same AHL production patterns, while identical AHL patterns were observed for strains ES213 and KB2B1, which appeared to be identical based on 3,170 core-genome genes (2). These results revealed the absence of a link between whole-genome phylogeny and AHL production patterns. AHL production patterns did not correlate with the sampling location of host squids (KB: ES114, KB4B5, and KB2B1 or MB: ES213 and MB13B1; 33); however, on a larger geographical scale (18), it has been demonstrated that despite the specificity between host and symbiont (27), symbiotic *V. fischeri* strains have the ability to infect genetically distinct hosts and evolve in order to accommodate to different host species (14, 31). Considering the importance of AHL production for the settlement of *V. fischeri* strains in the light organ of the squid, it seems possible to observe different AHL patterns between strains from distinct host species. Finally, AHL patterns coincided with three phenotypical traits: colony color, motility, and colonization capacities. Our results showed that oxo-C16:2-HSL is only observed in S-*type* strains (ES114, KB4B5, and MB13B1), which have reduced capacities to colonize the light organ, exhibited higher swimming rates and formed yellow colonies (34).

In the second step, we compared key proteins involved in AHL-mediated QS across our *V. fischeri* strains by focusing on the AHL synthases AinS and LuxI, the AHL membrane receptor AinR and the AHL intracellular receptor and transcriptional regulator LuxR. These four proteins were identified through *blastp* searches in strains ES213, KB2B1, MB13B1, and KB4B5 using the 4 proteins extracted from the ES114 genome (NCBI Accession number: NC_006840). First, we observed a clear separation between MB13B1 and the other strains based on AinS and LuxR phylogenies, but not for LuxI and AinR (Fig. 1 and S2). Such observation suggests that AHL production through the AHL synthase AinS as well as the signal reception by LuxR are likely to have substrate specificity, which may vary depending on the genetic diversity. Collins *et al*. (4) indicated that LuxR is evolutionarily pliable and promiscuous in its responses to AHLs with different acyl side chain lengths. However, regarding LuxR, we found that the difference between MB13B1 and the other strains was explained by 3 amino acid changes (Table 2 and Fig. S3), including 2 located in the amino terminal AHL-binding site, which has been shown to affect sensitivity to a broad spectrum of AHLs (4, 10). Previous studies identified the key amino acids for LuxR specificity towards the originally described signaling molecules, 3-oxo-C6-HSL and C8-HSL (4, 16); however, no information is currently available for the panel of AHLs described in the present study. AinS synthase has not yet been investigated in detail; however, Gilson *et al*. (11) demonstrated that the full-length AinS was necessary to produce AHL. This implies that the four amino-acid changes in MB13B1 can possibly have an impact on AHL synthesis, however the lack of structural information prevents any conclusions from being drawn (Table 2 and Fig. S4).

Remarkably, LuxI was found to be extremely conserved, with 100% identity between protein sequences among all *V. fischeri* strains, suggesting that signal synthesis has been maintained throughout evolution and that, in some way, it is important for individual fitness to keep this signal production active (3).

Recently, it has been demonstrated that the two AHL pathways LuxI/R and AinS/R are connected by an intracellular signaling cascade, and also that C8-HSL, produced by AinS, is able to bind LuxR and activate its own expression (8, 22, 23). In this context, LuxR must retain affinity for both AHLs (3-oxo-C6-HSL and C8-HSL), and possibly for all the AHLs produced by the two synthases (LuxI and AinS), in order to maintain the balance between the two circuits and regulate the different functions mediated by these autoinducers. In theory, if genetic modifications lead AinS to produce a slightly different set of AHLs, LuxR needs to “adapt” and co-evolve. In the case of MB13B1, these changes may have led to a decrease in sensitivity for 3-oxo-C6-HSL, which may explain why it produces larger quantities of this molecule. On the other hand, Kimbrough and Stabb (19) demonstrated that the complex LuxR/3-oxo-C6-HSL has the ability to bind an upstream region of the AinS/R locus and repress the expression of AinS. Assuming that C10-HSL and 3-OH-C12-HSL are produced by AinS, elevations in the production of 3-oxo-C6-HSL may have led to an increase in the complex LuxR/3-oxo-C6-HSL, the repression of AinS, and, if so, a decrease in C10-HSL and 3-OH-C12-HSL in MB13B1. In conclusion, the separation observed for AinS- and LuxR-based phylogenies tends to support that minor differences in their amino acid sequences might have led to slightly different “dialogues” among *V. fischeri* strains.

Our results suggest that AHL production through LuxI and signal reception by AinR might be evolutionarily stable, while AinS and LuxR exhibit variable signal synthesis and reception. Taking into account the unexpected diversity of AHL signaling molecules among symbiotic *V. fischeri* strains, it appears critical to understand which synthase, LuxI or AinS, is responsible for the synthesis of these molecules and the possible impact on the different components of the AHL-mediated QS pathways. Consequently, future studies are needed to complete the work of Colton *et al*. (5) on the sensitivity and the specificity of the two receptors (AinR and LuxR) towards all AHLs in order to clarify the role of multiple AHL signals in the physiology of *V. fischeri*.

## Supplementary Information



## Figures and Tables

**Fig. 1:**
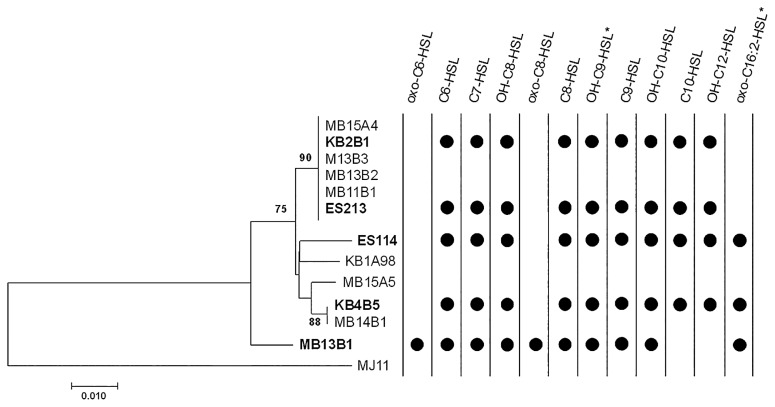
AHL production patterns in 5 symbiotic *V. fischeri* strains. Neighbor joining tree of the AinS sequences (355 aa) of 13 symbiotic *V. fischeri* strains using the JTT model (MEGA) (20). Bold characters indicate the strains used in the present study and asterisks show AHL structures that have not been confirmed with a corresponding standard. Bootstrap values are based on 1,000 replicates.

**Table 1:** Retention times for Cx-HSL, oxo-Cx-HSL, and OH-HSL. Predictions were based on the retention times curves of AHL standards (Fig. S1). NP: Non Predictable. Bold characters highlight the predicted retention times.

Acyl side chain length (C atoms)	Retention time (Rt, min)		

	Cx-HSL	oxo-Cx-HSL	OH-Cx-HSL
5	**7.85**	**6.97**	NP
6	8.43	7.56	NP
7	8.83	**8.15**	**8.09**
8	9.27	8.69	8.55
9	9.57	**9.04**	**8.92**
10	9.9	9.43	9.25
11	10.13	**9.74**	**9.59**
12	10.46	10.04	9.87
13	10.63	**10.33**	**10.15**
14	10.93	10.56	10.42
15	11.15	**10.83**	**10.63**
16	11.34	11.06	**10.85**
17	**11.49**	**11.27**	**11.05**
18	11.66	11.47	**11.24**

**Table 2:** Amino acid changes in *V. fischeri* MB13B1.

Protein	Amino acid changes	Property changes
**AinS**	Q42 → K	Amidic → Basic
	Q292 → K	Amidic → Basic
	S293 → A	Hydroxylic → Aliphatic
	T298 → A	Hydroxylic → Aliphatic

**LuxR**	T101 → V	Hydroxylic → Aliphatic
	D142 → N	Acidic → Amidic
	R176 → H	Basic → Basic
