# Supplementary material for: Evidence of a Large Diversity of N-acyl-Homoserine Lactones in Symbiotic Vibrio fischeri Strains Associated with the Squid Euprymna scolopes
Source: Microbes Environ. 2019 Feb 13;34(1):99–103. doi: 10.1264/jsme2.ME18145 (PMC6440727; doi:10.1264/jsme2.ME18145)

SUPPLEMENTARY INFORMATION

Table S1. UHPLC-HRMS data of AHL Standards. Rt: Retention time. Theoretical and Observed mass correspond to the pseudo-molecular ion $[M + H]^+$.

Standard	Molecular formula	Theorical mass	Observed mass	Rt (min)	Limit of detection (nM)
**C4-HSL**	C8H13NO3	172.0968	172.0968	5.27	>500
**C6-HSL**	C10H17NO3	200.1281	200.1281	8.43	3.64
**OXO-C6-HSL**	C10H15NO3	214.1074	214.1072	7.56	10.90
**C7-HSL**	C11H19NO3	214.1438	214.1440	8.83	5.33
**C8-HSL**	C12H21NO3	228.1594	228.1594	9.27	6.50
**OXO-C8-HSL**	C12H19NO4	242.1387	242.1381	8.69	6.15
**OH-C8-HSL**	C12H21NO4	244.1543	244.1540	8.55	-
**C9-HSL**	C13H23NO3	242.1751	242.1748	9.57	7.37
**C10-HSL**	C14H25NO3	256.1907	256.1907	9.90	4.56
**OXO-C10-HSL**	C14H23NO4	270.1700	270.1699	9.43	2.91
**OH-C10-HSL**	C14H25NO4	272.1856	272.1856	9.25	3.23
**C11-HSL**	C15H27NO3	270.2064	270.2063	10.13	9.11
**C12-HSL**	C16H29NO3	284.2220	284.2220	10.46	5.07
**OXO-C12-HSL**	C16H27NO4	298.2013	298.2013	10.04	21.28
**OH-C12-HSL**	C16H29NO4	300.2169	300.2169	9.87	2.39
**C13-HSL**	C17H31NO3	298.2377	298.2377	10.63	14.78
**C14-HSL**	C18H33NO3	312.2533	312.2533	10.93	11.82
**C14:1-HSL**	C18H31NO3	310.2377	310.2370	10.51	9.41
**OXO-C14:1-HSL**	C18H29NO4	324.2169	324.2170	10.23	4.71
**OXO-C14-HSL**	C18H31NO4	326.2326	326.2322	10.56	6.79
**OH-C14-HSL**	C18H33NO4	328.2482	328.2482	10.42	36.58
**C15-HSL**	C19H35NO3	326.2690	326.2689	11.15	15.11
**C16-HSL**	C20H37NO3	340.2846	340.2846	11.34	16.30
**C16:1-HSL**	C20H35NO3	338.2690	338.2704	10.93	14.68
**OXO-C16:1-HSL**	C20H33NO4	352.2482	352.2497	10.61	6.75
**C18-HSL**	C22H42NO3	368.3159	368.3155	11.66	10.48
**C18:1-HSL**	C22H39NO3	366.3003	366.3003	11.40	28.56

Table S2. UHPLC-HRMS data and identification of 3-OH-C9-HSL in *A. fischeri* strains. Rt: Retention time. Theoretical and observed mass correspond to the pseudo-molecular ion $[M + H]^{+}$. Bold characters in the fragmentation column are highlighting the daughter ions characteristic of the lactone ring.

Strain	Rt (min)	Observed mass	Molecular Formula	Delta ppm	Fragmentation	Identification			
						Name	Molecular Formula	Molecular Weight	Theorical Mass
**ES213**		258,1701		0,115	70.041 (16.93), **74.061 (9.53)**, 75.027 (16.64), 91.055 (14.94), 93.037 (31.41), **102.055 (12.41)**, 105.070 (10.50), 109.076 (66.51), 122.084 (18.27), 135.092 (53.91), 137.060 (14.71), 151.075 (81.97), 229.097 (23.28), 244.120 (21.08), 257.128 (13.45), 257.175 (16.47), 258.110 (21.03), 259.144 (100.00)				
**MB13B1**		258,1702		0,718	70.041 (10.26), **74.061 (9.68)**, 75.027 (12.38), 91.055 (7.87), 93.037 (16.40), **102.055 (35.76)**, 105.070 (6.38), 109.076 (31.04), 122.084 (10.24), 135.092 (52.27), 137.060 (8.18), 151.075 (33.32), 229.097 (23.46), 244.120 (23.04), 257.128 (6.53), 257.175 (6.67), 258.110 (9.27), 259.144 (100.00)				
**KB2B1**	8,9	258,1698	C13H24NO4	-0,185	70.041 (16.69), **74.061 (10.36)**, 75.027 (12.97), 91.055 (9.90), 93.037 (15.18), **102.055 (18.65)**, 105.070 (9.32), 109.076 (54.58), 122.084 (12.89), 135.092 (53.85), 137.060 (10.73), 151.075 (69.36), 229.097 (24.46), 244.120 (18.15), 257.128 (21.52), 257.176 (18.03), 258.110 (17.91), 259.144 (100.00)	OH-C9-HSL	C13H23NO4	257,1627	258,1700
**KB4B5**		258,1696		-0,395	70.041 (14.37), **74.061 (13.51),** 75.027 (18.06), 91.055 (12.21), 93.037 (29.99), **102.055 (20.07)**, 105.070 (12.15), 109.076 (56.22), 122.084 (12.35), 135.092 (45.94), 137.060 (10.86), 151.075 (72.83), 229.097 (22.02), 244.120 (18.48), 257.128 (23.68), 257.177 (14.71), 258.109 (18.25), 259.144 (100.00)				
**ES114**		258,1700		0,214	70.041 (9.43), **74.061 (10.47)**, 75.027 (36.97), 91.055 (8.93), 93.037 (100.00), **102.055 (14.35)**, 105.070 (9.00), 109.076 (45.02), 122.084 (9.23), 135.092 (76.51), 137.060 (9.31), 151.075 (51.00), 229.097 (29.82), 244.120 (11.06), 257.128 (15.74), 257.176 (13.72), 258.110 (22.61), 259.144 (100.00)				

Table S3. UHPLC-HRMS data and identification of oxo-C16:2-HSL in *A. fischeri* strains. Rt: Retention time. Theoretical and observed mass correspond to the pseudo-molecular ion $[M + H]^+$. Bold characters in the fragmentation column are highlighting the daughter ions characteristic of the lactone ring.

Strain	Rt (min)	Observed mass	Molecular Formula	Delta ppm	Fragmentation	Identification			
						Name	Molecular Formula	Molecular Weight	Theorical Mass
**ES114**	9,97	350,2326	C20H32NO4	-0,043	**56.050 (2.68)**, 57.071 (19.57), 69.071 (3.46), 71.086 (24.65), 73.066 (3.34), **74.061 (13.01)**, 89.060 (75.83), 95.086 (0.69), **102.055 (17.33)**, 105.070 (100.00), 106.074 (7.71), 109.102 (2.31), 123.085 (1.89), 127.112 (9.03), 133.086 (17.83), 146.081 (6.71), 224.128 (17.40)	OXO-C16:2-HSL	C20H31NO4	349,2253	350,2326
**KB4B5**		350,2324		-0,557	**56.050 (1.26)**, 57.071 (22.15), 69.071 (4.62), 71.086 (32.15), 73.066 (2.63), **74.061 (10.98)**, 89.060 (55.17), 95.086 (2.30), **102.055 (17.11)**, 105.070 (100.00), 106.074 (7.70), 109.102 (3.48), 123.081 (2.07), 127.112 (10.10), 133.086 (12.62), 146.081 (3.12), 224.128 (12.15)				
**MB13B1**		350,2325		-0,302	**56.050 (3.02)**, 57.071 (21.38), 69.071 (4.10), 71.086 (17.95), 73.066 (6.71), **74.061 (21.54)**, 89.060 (38.94), 95.086 (1.56), **102.055 (19.36)**, 105.070 (100.00), 106.074 (6.27), 109.102 (4.63), 123.081 (1.93), 127.112 (12.08), 133.086 (19.49), 146.081 (4.58), 224.128 (15.72)				

Fig. S1. Retention times curves for Cx-HSL, oxo-Cx-HSL and OH-Cx-HSL based on 23 AHL standards (R^2 >0.99).

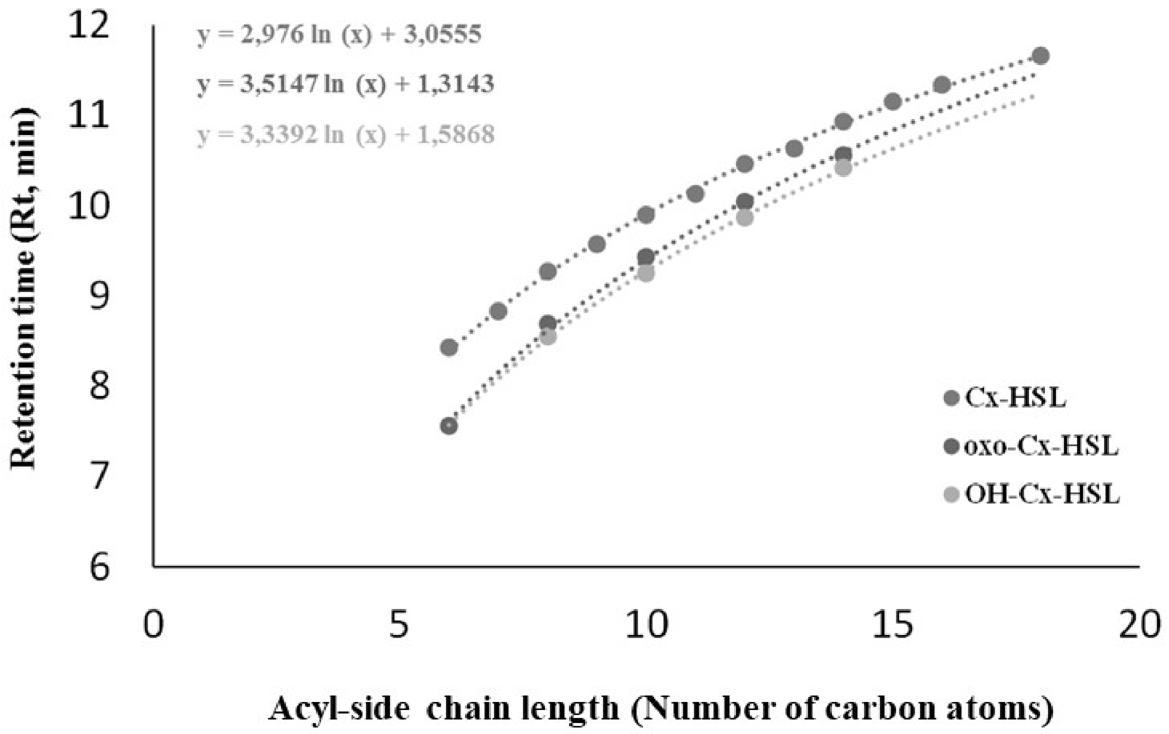

Fig. S2. Neighbour joining tree of 13 symbiotic *V. fischeri* strains using the JTT model (MEGA). Bootstrap values are based on 1000 replicates. **A**: AinR (623 aa); **B**: LuxI (190 aa) and **C**: LuxR (250 aa).

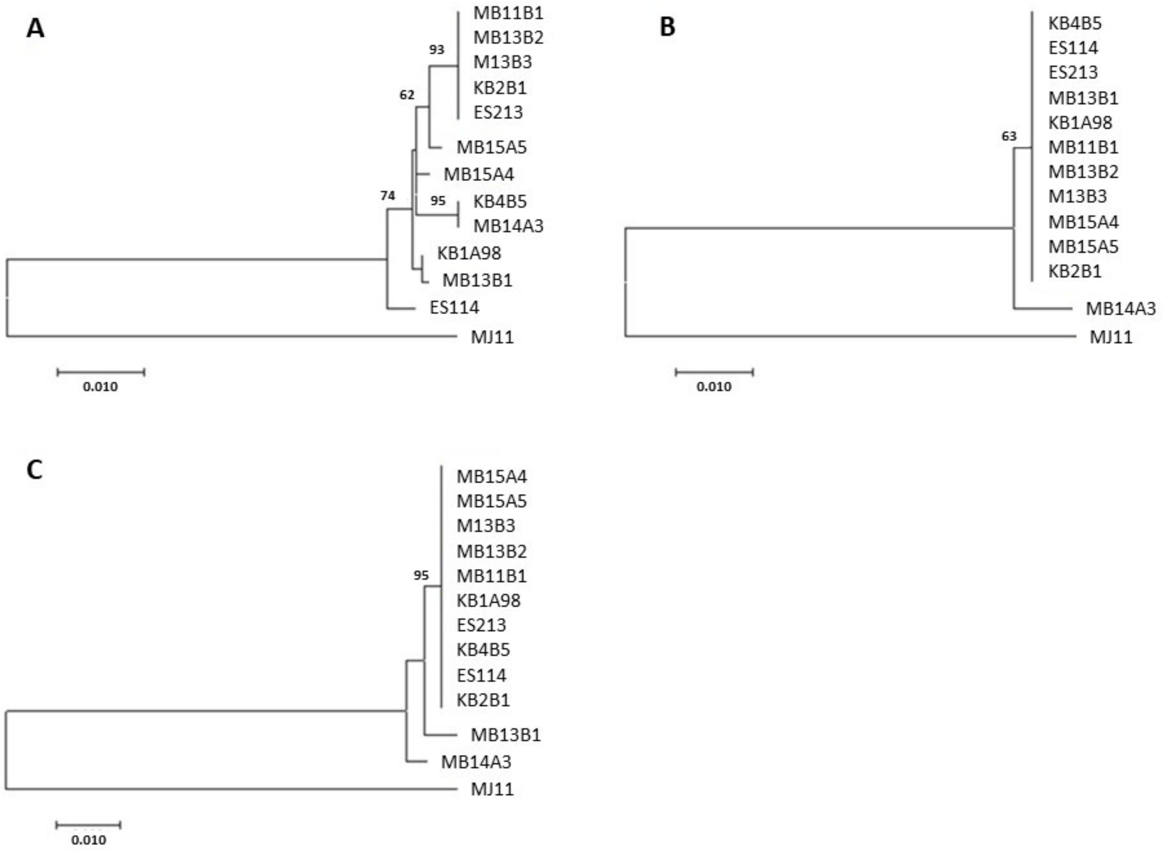

Fig. S3. Alignment of LuxR proteins from the 5 *V. fischeri* strains (250 aa). Asterisks are indicating the amino acid changes in MB13B1 described in Table 2.

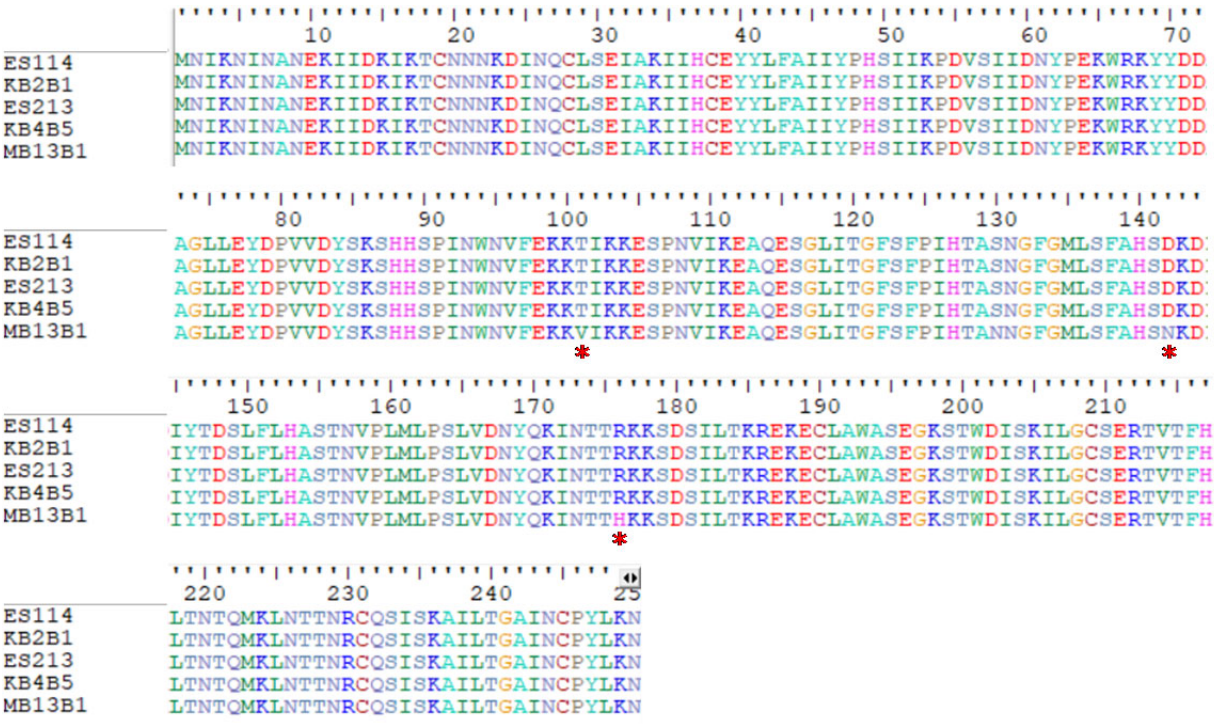

Fig. S4. Alignment of AinS proteins from the 5 *V. fischeri* strains (355 aa). Asterisks are indicating the amino acid changes in MB13B1 described in Table 2. Accession numbers: ES114: WP_011261670.1; ES213/KB2B1: WP_081248857.1; KB4B5: WP_081249183.1; MB13B1: WP_081249608.1.

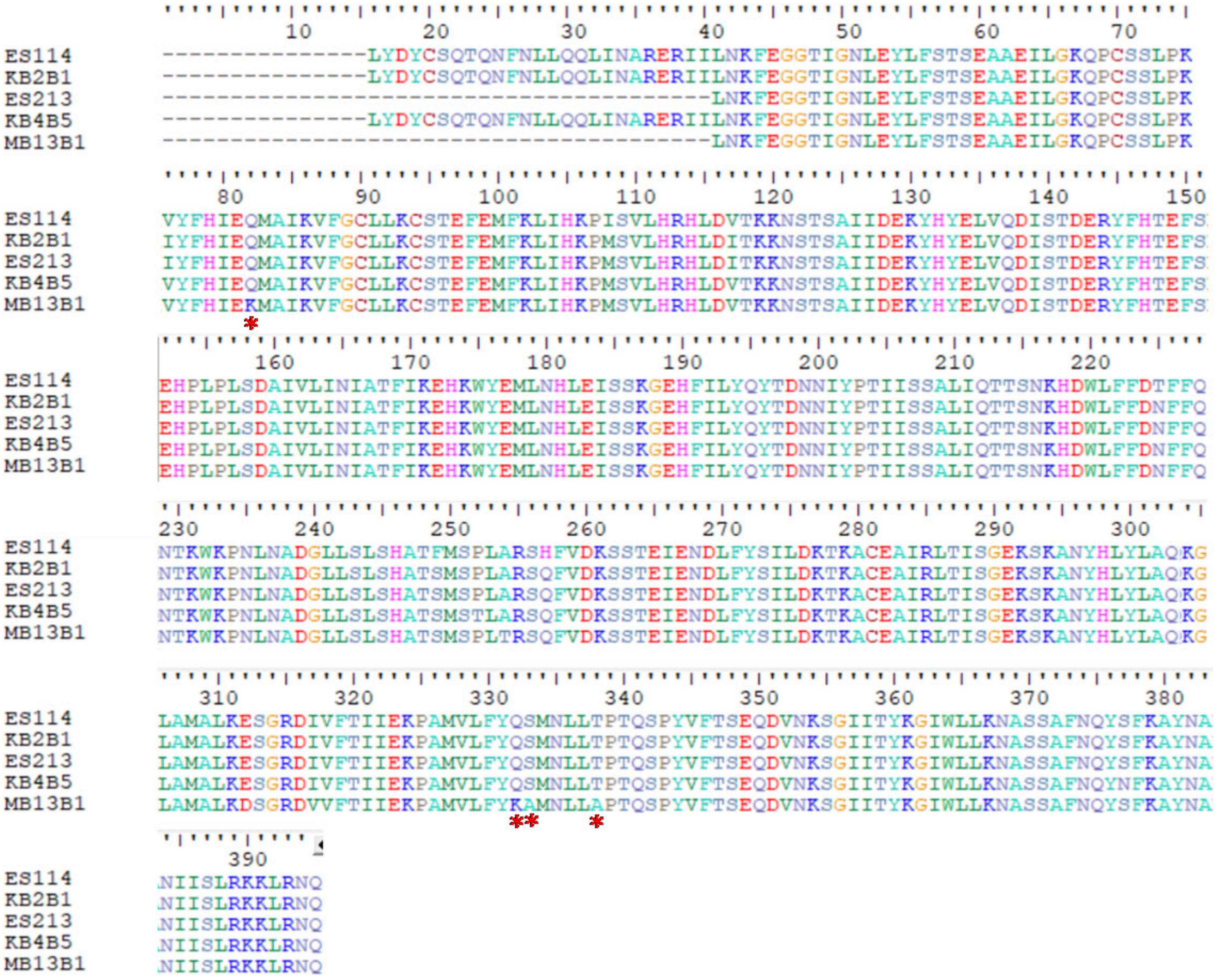

Supplement: Supplementary file 1 [file 34_99_s1.pdf]
